# The Evolution of Human Handedness

**DOI:** 10.1111/nyas.12102

**Published:** 2013-04-30

**Authors:** I C McManus, Angus Davison, John A L Armour

**Affiliations:** 1 Research Department of Clinical, Educational and Health Psychology, University College LondonLondon, United Kingdom; 2 School of Biology, University ParkNottingham, United Kingdom; 3 School of Biology, Queen's Medical Centre, University of NottinghamNottingham, United Kingdom

**Keywords:** handedness, inheritance, genetics, GWAS, multilocus model, family data

## Abstract

Right- and left-handedness run in families, show greater concordance in monozygotic than dizygotic twins, and are well described by single-locus Mendelian models. Here we summarize a large genome-wide association study (GWAS) that finds no significant associations with handedness and is consistent with a meta-analysis of GWASs. The GWAS had 99% power to detect a single locus using the conventional criterion of *P* < 5 × 10^−8^ for the single locus models of McManus and Annett. The strong conclusion is that handedness is not controlled by a single genetic locus. A consideration of the genetic architecture of height, primary ciliary dyskinesia, and intelligence suggests that handedness inheritance can be explained by a multilocus variant of the McManus *DC* model, classical effects on family and twins being barely distinguishable from the single locus model. Based on the ENGAGE meta-analysis of GWASs, we estimate at least 40 loci are involved in determining handedness.

## The genetics of handedness

Right- and left-handedness show many features that suggest that they are under genetic control. For example, left-handedness runs in families,^1^,^2^ and monozygotic (MZ) twins are more concordant than those that are dizygotic (DZ);^2^ there is also little compelling evidence of environmental factors, and the scarce data from adoption studies are compatible with genetic effects.^3^

## Single-locus models

Most models of the inheritance of handedness, from the early model of Ramaley^4^ through to the models of McManus,^5^ Annett,^6^ and Klar,^7^ describe a single locus with two or more alleles; fewer models specify two loci.^8^ For simplicity, this paper mainly considers the McManus *DC* model, which describes additive expression at a single locus with two alleles, *D* (Dextral) and *C* (Chance), and the three genotypes being *DD*, *DC*, and *CC*, although most of the conclusions broadly and similarly apply to other genetic models. In the *DC* model, the probabilities of left-handedness given the three genotypes, *P*(*L*|*DD*), *P*(*L*|*DC*), and *P*(*L*|*CC*), are 0%, 25%, and 50%. For a typical population rate of left-handedness, *P*(*L*) = 10%, and for the frequency of the *C* allele, *P*(*C*) = 20%. The McManus model, as well as the Annett model, is successful in its predictions because the *C* allele (or Annett's *RS*-allele) produces randomness in the phenotypes. The phenotype of the *CC* genotype with its 50:50 mixture of right- and left-handedness does not indicate remaining genetic or environmental variance, but instead the phenotype itself is what can be called *deep chance*, corresponding to fluctuating asymmetry in biology;^9^ in effect, ineradicable random biological noise. A good biological example of such a process in mice is the *iv* gene,^10^ in which wild-type mice (+/+), as well as heterozygotes (*iv*/+), show *situs solitus* (ss), where the normal position of the heart is on the left, and the other viscera are in their usual lateral locations (e.g., liver to the right, spleen to the left). However, *iv* homozygotes (*iv/iv*) manifest as 50% with *situs solitus* and 50% with *situs inversus* (si; heart on the right, liver on the left, spleen on the right). The absence of remaining genetic variation is shown because crosses between *iv/iv* mice produce offspring that are independent of parental phenotypes *iv/iv* × *iv/iv* and parental pairs, which are phenotypically ss × ss, ss × si, or si × si, all producing 50% si offspring.

## Family data

Handedness in families and twins shows several unusual features, which the McManus model (and also the Annett model) account for successfully. Typical rates of left-handedness in Western societies are about 10–12%, although there are geographical and historical variations.^11^ A value of 10% is convenient for exposition. Neither right- nor left-handedness breeds true in families since *R* × *R* parents have some left-handed offspring, and *L* × *L* parents have, in fact, mostly right-handed offspring (a combined figure from 25 studies giving an overall estimate of 26% of 417 offspring^1^). The McManus model explains this: right-handers can be *DD*, *DC*, or *CC*, and the right-handers who are *DC* or *CC* can have left-handed offspring, 7.8% overall being predicted to be left-handed. Left-handers can only be *DC* or *CC*, and the majority will be *DC. DC* × *DC* parents will produce 25% left-handed offspring and, overall, only 30% of the offspring of two left-handed parents will be left-handed (meaning the majority of offspring are right-handed).

## Twins

Handedness in MZ twins has traditionally been problematic for genetic models because of the high rate of discordance. A meta-analysis,^12^ in which 12.74% of MZ twins were left-handed, found that 19.3% of the 10,001 twin pairs were discordant for handedness (R–L), a rate significantly lower than that for DZ twins, whose discordance was 20.3%. Monozygotic discordance is explained by *DC* and *CC* twins each having a 25% or 50% probability of left-handedness, those probabilities being independent in the ontogeny of each twin. Therefore, for *CC* MZ twins, 0.25 will be R–R, 0.25 will be L–L, and 0.5 will be R–L, while for the more frequent *DC* MZ twins, the proportions of R–R, R–L, and L–L pairs are 9/16, 6/16, and 1/16. Overall, for 10% population left-handedness, the McManus model predicts that 14% of MZ pairs are discordant, a lower rate than the 16% predicted for DZ twins. Discordant pairs should also be more frequent in twins with left-handed parents^13^ (unpublished data, personal communication, I.C. McManus, A. Davidson, J.A. Armour, November 1996). Ultimately, genetic models do not predict that MZ twin pairs should all be concordant, but that the rate of concordance should be higher in MZ than DZ twins, as indeed is the case for handedness.

## Language lateralization

Handedness correlates with key human cognitive processes of language, which, as Dax and Broca showed in the 19th century, are mainly carried out by the left hemisphere of the brain.^14^ Although left-handers are often assumed to have right-hemisphere dominance for language, that is mostly not the case, as 1 in 20 right-handers and one in three left-handers have language functions that involve processing by the right hemisphere.^15^,^16^ Most left-handers therefore have left-hemisphere language-processing functions, like right-handers, and individuals with right-hemisphere language functions are therefore about equally likely to be right- or left-handed. In brief, this pattern is readily explained if language lateralization is pleiotropically determined by the *D* and *C* alleles; the *DC* and *CC* genotypes having a 25% and 50% probability, respectively, of right-language lateralization, with this probability being independent of the probability of being right- or left-handed. The result is that 7.8% of right-handers and 30% of left-handers would be expected to have right-language dominance, which fits well with the data.

## Finding the gene for handedness

Since handedness appears to be inherited at a single locus, it might be expected, in an age of molecular genetics, that finding the gene should be straightforward. However, the combination of additive inheritance and the randomness resulting from fluctuating asymmetry substantially reduces the power of standard methods, such as linkage and association. The advent of large-scale genome-wide association studies (GWASs) should change that, as long as sample sizes are sufficient. Because there are only a small number of published GWASs looking at handedness (which are reviewed below), data are analyzed here from a large molecular genetic study in order to look for associations with handedness on a genome-wide basis. These results will be presented in detail in a separate publication,^17^ but a simple summary is all that is needed for present purposes: not one of the single-nucleotide polymorphisms (SNPs) analyzed reached the critical level of 5 × 10^−8^.

## Power calculations

Although our GWAS analysis found no significant associations, a key question is whether those analyses are compelling evidence against a single gene for handedness, or whether perhaps there was simply insufficient power to convincingly be able to come to a negative conclusion. Formal power calculation was therefore carried out, given our sample size of about 3750 individuals, to assess the likelihood of finding an association with a single-locus gene. To ensure that a single locus could be rejected by such data, we set the power level at 99%.

Although it is possible to consider the problem of power analytically (and this basic approach has been described elsewhere^18^), any such method does have problems, not least because SNPs are not distributed evenly across the genome, minor allele frequencies (MAFs) differ across SNPs, and the particular mapping of SNPs on the genome depends on the sequencing chip that has been used. Fortunately, a more formal calculation can be carried out.^19^ Although full details will be presented elsewhere, our power calculations used 10 million Monte Carlo simulations. The conventional significance level for a GWAS is 5 × 10^−8^, calculated as reflecting the usual *P* < 0.05 α criterion, Bonferroni-corrected for 1 million possible loci (based on 3 billion bases and a possible gene length of 3000 bytes). On that basis, power for the standard McManus model in our GWAS was 99.47% (and 99.17% for the Annett model), adequate power for detecting a single locus.

## Molecular genetic studies of handedness: a review

Many molecular studies have searched for autosomal genes associated with handedness (Table 1). Interpretating these data can be challenging since some studies use difference scores on a peg-moving task, which can correlate with overall motor proficiency and is only weakly associated with hand preference. What is clear is that no associations replicate across studies, with significance levels often being marginal after correction for multiple testing, and several studies find no significant associations at all (Table 1). The present review does not discuss specific sex-chromosomal genes, but there are claims for the importance of the sex-specific, nonrecombining, X–Y homologous genes *protocadherin X* and *protocadherin Y*,^20^–^22^ and for variation in CAG repeat length in the androgen receptor.^23^ Given undoubted sex differences in the prevalence of left-handedness,^24^,^25^ these genes may moderate handedness,^1^ although handedness certainly is not inherited as a classic, sex-linked Mendelian gene.^26^

**Table 1:** Summary of molecular studies of the genetics of handedness

Study and date	Participants and method	Handedness/lateralization criteria	Significance levels	Chromosome	Genes/SNPs
Laval *et al*.^20^	180 pairs of left-handed brothers recruited through the media. 14 genetic markers spanning the X-chromosome	Annett Handedness Questionnaire and pegboard	No evidence for a locus linked to increased likelihood of left-handedness. One locus related to relative hand skill (nominal *P* < 0.002)	X	DXS990 in Xq13
Van Agtmael *et al*.^47^	Study of six candidate genes (*DNAHC1*, *DNAHC6*/*DNAHC8*, *LRD*, *NODAL*, *DNAHC13*, *DNAHC2*) in one large, extended pedigree and 27 nuclear families. *n* = 173	Edinburgh Handedness Inventory	No associations reported as significant for either the McManus or the Klar model	n/a	n/a
Francks *et al*.^48^ (and erratum^49^)	89 nuclear families with at least two siblings with dyslexia (*n* = 195). 401 polymorphic markers. Independent replication sample, *n* = 143 sibling pairs, 11 markers on 2p11.2–12	Pegboard asymmetry calculated as (*L*−*R*)/((*L*+*R*)/2)	No markers achieved the critical level of *P* = 0.00002., but 2p11.2–12 had a level of *P* = 0.00007, which the authors called “a putative QTL.” The replication sample had *P* = 0.13	2	Region 2p11.2–12
Francks *et al*.^50^	New sample of 105 pairs of brothers, previously analyzed by Laval *et al*.^20^ Seven microsatellites spanning 2p16–q14	Pegboard asymmetry calculated as (*L*−*R*)/((*L*+*R*)/2)	*P* = 0.00035, which exceeded the critical value of *P* = 0.01	2	Maximum linkage at 2p12–q11
Francks *et al*.^51^	Reanalysis of previous data from Ref. 48. 87 SNPs within 2p12–p11, targeting four genes (*LRRTM4*, *CTNNA2*, *LRRTM1* and *DNAH6*). Replication study using 354 sib-pairs from 215 Australian twin families	Pegboard asymmetry calculated as (*L*−*R*)/((*L*+*R*)/2). Criteria for handedness in the Australian sample not documented	Strong paternal association in the main sample with the imprinted gene *LRRTM1*. No association in the twin replication study (*P* > 0.1)	2	*LRRTM1* (Leucine-rich repeat transmembrane neuronal 1)
Warren *et al*.^52^	584 participants in primary study of gallbladder disease. 382 markers at ∼10cM intervals	Short form of Edinburgh Handedness Inventory, including items on eyedness and footedness	No markers were associated significantly with phenotypes using a criterion of LOD ≥ 3	n/a	n/a
Engage Handedness Consortium^12^	Meta-analysis of 12 (unstated) GWASs, based on 2350 left-handers and 21093 controls. 2.5 million SNPs and imputed SNPs	Writing hand	No SNPs reached criterion of 5 × 10^−8^; highest signals were 4 × 10^−7^ and 6.15 × 10^−7^	7, 13	*SLIT3* (axon-guidance-pathways)*MAB21L1* (cerebellar development)*NBEA* (neuron-specific protein)
Eriksson *et al*.^53^	Web-based survey of 23andMe customers. *n* = 4268 for handedness. 580,000 SNPs	Annett Handedness Questionnaire; Waterloo Footedness Inventory; eyedness and hand-clasping	Largest significance level for handedness was 5.0 × 10^−6^; no significant associations either for footedness, eye dominance or hand clasping	n/a	n/a
Scerri *et al*.^54^	Stage 1: 192 individuals from families with reading disorder, analyzed previously;^48^Illumina 550k SNP array and 2 million imputed SNPs. Stage 2: Replication sample of 368 individuals with reading disorder. Stage 3: 185 children from the Avon longitudinal study with reading disability	Pegboard asymmetry calculated as (*L*−*R*)/((*L*+*R*)/2)	Stage 1: No SNPs were significant with *P* < 5 × 10^−8^. Strongest signals were *P* = 4.7 × 10^−7^ for rs11855415, and 1.1 × 10^−6^ for rs9806256. Stage 2: rs 1185415, *P* = 0.033; rs9806526, *P* = 0.18. Stage 3: rs 1185415, *P* = 0.0025; rs9806526, *P* = 0.00067. Meta-analytic result: rs 1185415, *P* = 0.1.99 × 10^−8^; rs9806526, *P* = 2.34 × 10^−7^	15	*PCSK6* (proprotein convertase subtilisin/kexin type 6), thought to be involved in left–right axis formation
Armour *et al*.^17^	See the main text for description of this study	Writing hand	No SNPs reached the criterion of *P* < 5 × 10^−8^	n/a	n/a

The European Network for Genetic and Genomic Epidemiology (ENGAGE) consortium reported meta-analysis of 12 GWASs, based on 2350 left-handers and 21,093 controls. No associations reached conventional GWAS significance, although three approached it, and it was said that, “large-scale replication effects are currently underway.” Since then, in 2010, the International Handedness Consortium reported data from 5429 left-handers and 49,970 right-handers from 32 separate GWASs,^27^ but with evidence of heterogeneity between the GWASs. Given such vast sample sizes, and the clear power of our own much smaller GWAS, a fortiori there must have been more than adequate power in those much larger databases of the International Handedness Consortium.^12^,^27^ These studies clearly suggest that there is no single autosomal locus for handedness.

Handedness is far from being the only common trait where problems have arisen in finding genetic associations. As Crow has said for psychiatric conditions, where once there was “widespread optimism [that …] all that was necessary was to ‘drain the pond dry’ to reveal the relevant genes”^21^ (p. 319), a much more critical approach is now being adopted. The largest extant GWAS is for height,^28^ which, with 183,727 individuals, did identify 180 loci accounting for at best 20% of variance, despite height having a very high conventional heritability. In a critical review of the genetic architecture of psychiatric genetics in particular,^29^ it was suggested that the genetic architecture of complex psychiatric traits may well resemble that of height, not least on evolutionary grounds. The review cited the analyses of Eyre-Walker, who says that, for complex conditions evolving under selection, “most of the variance in fitness is contributed by mutations of large effect that are very rare in the population”^30^ (p. 1755), a conclusion similarly made by others.^31^ It is not surprising that GWASs have problems, and equally unsurprising that handedness has similar problems.

## Implications for genetic models of handedness

The large GWASs reported here had adequate power to detect a single major gene determining handedness. Even if no single locus accounts for variation in handedness, it does not mean that handedness is not under genetic control. Before looking at handedness in particular, the findings of GWASs will be considered first for understanding traits and conditions such as intelligence and primary ciliary dyskinesia.

## Primary ciliary dyskinesia

A key model for thinking about handedness is primary ciliary dyskinesia (PCD; Kartagener's syndrome), which results in chronic bronchiectasis and sinusitis, infertility, and, in 50% of cases, si.^32^ The fundamental defect is of normal ciliary motility, which, for the laterality defect, is in the nodal region of the embryo.^33^ Although PCD is familial, a GWAS found strong evidence of locus heterogeneity,^34^ and at least 16 different loci causing PCD have currently been identified^35^–^37^ (Hannah Mitchison, personal communication, 2012). Motile cilia are extremely complex biomolecular machines^38^ (see http://www.ciliaproteome.org), with recent estimates suggesting well over 800 proteins in the ciliary proteome (Hannah Mitchison, personal communication, 2012), disruption of any of which might result in dysfunction. It is not surprising that many disease loci have been found despite the broad phenotype being pathophysiologically similar across cases.

## Intelligence

Although about half of the variance in intelligence is under genetic control,^39^ large-scale GWASs found no single gene associated with intellectual ability.^40^ A different approach, Genome-wide Complex Trait Analysis (GCTA), which assesses how all SNPs jointly contribute to phenotypic variation,^41^ strongly supports classical polygenic models, with 40–50% of variance due to the accumulated effect of large numbers of SNPs.^40^ Intelligence therefore seems to be determined by large numbers of genes, mostly with small effects, although those individual genes run in families, so correlations exist between twins and other family members.

## A pathology model

Both PCD and intelligence can be construed as pathology models, with variance mostly caused by rare, recent, deleterious genes.^31^ That is writ large for the brain, but is similar for PCD. Complex biological machines (cilia, the brain) require the integrative functioning of multiple processes dependent on genes. Mutations mostly result in damage to the components, which can accumulate and produce larger changes. For brain function, many genes run in families, which produce brains that function well or less well. Mutations resulting in better functioning brains are necessarily rare, but selection, as presumably occurred in human evolution with its large increases in brain size and intelligence, probably takes those genes to fixation, so they contribute little to no variance (although different interpretations exist^42^).

## The genetic architecture of handedness

Our within-families analysis suggests that there is no single gene for handedness. However, our GWAS and the meta-analysis of Medland *et al*.^12^ exclude most single-locus models. That is parallel to the approach taken by studies on intelligence, although there is currently no GCTA that would clinch the conclusion that handedness is under genetic control despite the absence of association in a GWAS. Nevertheless, it is necessary to consider multilocus models.

### A multilocus version of the McManus model

The *DC* model can readily be reconceptualized with *n* loci, where *n* can be from two to some large number. At the *i*th locus, let there be alleles conceptually equivalent to *D* and *C* in the single-locus model, which are labelled *D_i_* and *C_i_* (*i* = 1:*n*). For simplicity, assume that the frequencies of the C*_i_* alleles, *c_i_*, are equal (although relaxing this assumption makes little difference to the model^a^). Since *C_i_* alleles are rare (Table 2), individuals will be homozygous at most loci for the *D_i_* allele (*D_i_D_i_*). Individuals with only *D_i_* alleles at all loci are equivalent to *DD* individuals in the single-locus model, and all will be right-handed. Similarly, individuals with two *C_i_* alleles at just one locus (*C_i_C_i_*) are 50% left-handed, as are *CC* individuals in the single-locus model, and individuals with a single *C_i_* allele (i.e., *D_i_C_i_* at one locus) are 25% left-handed. Using a damage model akin to that in PCD, if being homozygous for *C_i_* at a single locus produces fluctuating asymmetry by preventing ciliary action, then being homozygous at two or more loci will have no greater effect, and hence individuals who are *C_i_C_i_* at multiple loci will also show 50% left-handedness. Likewise, individuals who are heterozygotes at multiple loci (*D_i_C_i_*) will have a 25% chance of left-handedness.

**Table 2:** Predicted handedness in families and in monozygotic twins for the single-locus model (*N* loci = 1) and multilocus models^a^

		Percent left-handedness by parental phenotype			Percent concordance and discordance in monozygotic twins		
*N* loci	*c_i_*	*R* × *R*	*R* × *L*	*L* × *L*	*R – R*	*R – L*	*L – L*
1	*0.20000*	7.82%	18.90%	30.63%	83.00%	14.00%	3.00%
		(7.78%)	(18.89%)	(30.00%)	(83.00%)	(14.00%)	(3.00%)
2	*0.11110*	8.15%	17.74%	25.56%	82.80%	14.40%	2.83%
3	*0.07715*	8.19%	17.24%	24.17%	82.74%	14.55%	2.71%
4	*0.05916*	8.29%	17.01%	22.88%	82.70%	14.64%	2.66%
5	*0.04780*	8.35%	16.79%	22.60%	82.65%	14.69%	2.66%
10	*0.02473*	8.38%	16.45%	21.86%	82.60%	14.85%	2.55%
20	*0.01256*	8.46%	16.50%	21.53%	82.50%	14.95%	2.55%
50	*0.00507*	8.52%	16.09%	20.02%	82.51%	14.97%	2.53%
100	*0.00254*	8.48%	16.10%	20.28%	82.52%	14.98%	2.49%
200	*0.00127*	8.55%	16.30%	20.83%	82.39%	15.09%	2.51%
500	*0.00051*	8.56%	16.12%	21.06%	82.54%	14.91%	2.55%
1000	*0.00026*	8.52%	16.26%	20.29%	82.46%	15.03%	2.51%
Approximate CI		±0.05%	±0.07%	±0.08%	±0.08%	±0.07%	±0.03%

a Results are from 1,000,000 Monte Carlo simulations for *p*(*L*) = 10%, with the exception of values in parentheses for *N* loci = 1, which are analytic solutions. For Monte Carlo calculations, approximate 95% confidence intervals (CI) are shown in the last row.

### Calculations

For the *i*th locus, the probability of being *D_i_D_i_* is (1− *c_i_*),^2^ so the probability of being *D_i_D_i_* for all loci is (1− *c_i_*)^2*n*^. Likewise, the probability of a single locus being *C_i_C_i_* is *c_i_*^2^, and, therefore, the probability of having no *C_i_C_i_* genotypes is (1 − *c_i_*^2^)*^n^*; hence, the probability of having at least one *C_i_C_i_* genotype is 1 − (1 − *c_i_*^2^)*^n^*. The probability of having at least one *D_i_C_i_* genotype is then 1-[1- (1− *c_i_*^2^)*^n^*] − (1− *c_i_*^2^)*^n^* = (1− *c_i_*^2^)*^n^*− (1−*c_i_*)^2*n*^. Since one quarter of *D_i_C_i_* and one half of *C_i_C_i_* are left-handed, then


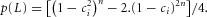


This equation can then be solved for *c_i_* for any value of *p*(*L*); see Table 2 for values of *p*(*L*) = 10% and *n* from 1 to 1000.

### Families and twins

Analytic solutions for varying numbers of multiple loci are possible, but for ease we have resorted here to a Monte Carlo simulation (Table 2 showing multiloci models for a range of values of *n*). The heritability of handedness does decrease slightly with more loci, *R* × *R* parents having slightly more left-handed offspring and *L* × *L* parents having slightly fewer left-handed offspring. Those differences arise because, although 50% of offspring from two *CC* parents (*CC* × *CC*) would be left-handed in the single-locus case, with multiple loci, the *C_i_* alleles may be at different loci (e.g., *D_i_C_i_*; *D_j_C_j_* × *D_p_C_p_*; *D_q_C_q_*), producing only 25% of left-handedness. For MZ twins, the increasing numbers of loci also result in a slightly higher rate of discordant pairs.

### Distinguishing single- and multilocus models

Since the differences between multi- and single-locus models are small (Table 2), these models would be hard to distinguish from each other using classical genetics with reasonable sample sizes. The multilocus model is effectively indistinguishable from the single-locus model for small to medium sample sizes, as distinguishing 7.82% from 8.52% of left-handers in the offspring of *R* × *R* parents with a power of 80% would require 24,034 parent–offspring pairs. Similarly, distinguishing 14% from 15.03% of discordant MZ twins would require 18,359 pairs, which is outside the range of most studies. It can broadly be concluded that single- and multilocus models make equivalent predictions for families and twins. Although the assumption was made that all *c_i_* are equal, that assumption can readily be relaxed*^a^* and predictions do not differ particularly unless one *c_i_* is very large. If the single- and multilocus models are effectively indistinguishable in terms of classical genetics, then the only approach that is likely to have adequate power to compare them is whole exome or whole genome sequencing in large families, searching for particular mutations associated with handedness, with the expectation of *n* such genes, with different alleles in different families. A consortium of researchers, including one of us (I.C.M.), has submitted an application to carry out such a study.

### Power for detecting multiple loci for handedness

In our own GWAS, the power for detecting a locus was calculated if there were 10 loci at about 85%. Calculation of power for the much larger ENGAGE consortium data, which had an *n* of 23,443, is much more computationally intensive. ENGAGE was estimated to have a power of 99.26% to detect a McManus model with 30 equi-frequent loci, and 98.96% with 40 loci. Although power for larger numbers of loci was lower, it was still about 96% with 100 equi-frequent loci. The much larger sample size of over 55,000 in the meta-analysis of the International Handedness Consortium (see above) will have still larger power. It seems reasonable to conclude, given the lack of any significant results in the ENGAGE analysis, that there are probably at least 30–40 loci involved in handedness, or a significant result would have probably been found.

## The biological mechanisms underpinning handedness

Little is known about the nature of the biological mechanisms underpinning handedness/cerebral lateralization (although McManus^24^,^43^ speculated that it may have involved duplication and modification of mechanisms originally responsible for *situs* lateralization). Primary ciliary dyskinesia might provide a useful model for the type of mechanism we are proposing for handedness, with multiple genes being affected. It must, however, be stressed that the genes for primary ciliary dyskinesia are not the same as those for handedness, since handedness and *situs inversus/solitus* in PCD are not associated;^44^ left-handedness shows the same rates in PCD-si, PCD-ss, and the general population. That constraint does not exclude the possibility that a separate but different ciliary or other mechanism exists for determining brain asymmetries rather than visceral asymmetries, so that mutations will affect the two systems independently. Alternatively, there must be some other, as yet unknown, mechanism for determining brain asymmetries. As long as that mechanism involves a chain or cascade of processes, or some form of molecular machinery with multiple interacting components, then damage at many possible points (i.e., multiple genetic loci) could result in failure of the mechanism, and hence left- rather than right-handedness.

## The selective advantages and disadvantages of left-handedness

Consideration of the details of underlying biological mechanisms that determine right-handedness is far from straightforward, not least because there are strong reasons to believe that there must be at least some selective advantages to being left-handedness or genetic drift would have resulted in fixation of one or other allele, resulting in a population either of right-handers (all *DD*), or a racemic mixture of right- and left-handers (all CC). The most likely possibility for maintenance of left-handedness with a single-locus model is a heterozygote advantage where *DC* genotypes having greater fitness. Similar selective pressures could in principle work in parallel on all of the *C_i_* alleles (or, more particularly, the *D_i_C_i_* genotypes, which would be advantaged). It has been speculated that the benefits of *DC* individuals come from partial randomization resulting in cerebral polymorphisms,^11^ some of which give processing advantages due to cognitive modules, which are normally in separate hemispheres and occasionally colateralized within a hemisphere. For most purposes, that mechanism would not be altered by a multilocus model of the type described here.

Frequency-dependent selection is also possible, although that is more complicated, not only because selection can either be purely at the level of the handedness phenotype (i.e., right and left; in which case it would not matter which of the *C_i_* alleles was involved in creating the phenotype), or, in effect, it could be at the level of the genotype,^45^ with *DD*, *DC*, and *CC* all having differing phenotypes because of cerebral polymorphism. (In this case, the frequency-dependent selection would presumably occur separately at each of the loci, and would change the dynamics as the separate *C_i_* alleles would individually be much more rare.) More detailed modeling of frequency-dependent selection has been carried out in both of these terms, and with differing degrees of balanced polymorphism (unpublished observations, I.C. McManus). Again, the main conclusion is that single- and multilocus models do not differ in any substantive way.

## The separate evolution of right-handedness and left-handedness

Although current human populations are polymorphic for handedness, this may not necessarily have been the case during the evolution of *Homo sapiens*. As discussed elsewhere,^43^ the *ur-*state for the last common ancestor of humans and chimpanzees/bonobos could well have been a 50:50 mix of right- and left-handers. The next step need not, however, have been the present 90:10 mix of right- and left-handers. If the *D_i_* alleles allowed brain asymmetry, right-handedness, language and tool use, then they could rapidly have run to fixation, the population consisting entirely of right-handers (100:0). The erudite and convincing theory of Crow shows how events on the X and Y chromosomes may have been responsible for such a saltatory, species-producing event^22^,^46^ in which the entire population was right-handed. Left-handedness (and presumably, the modern *C_i_* allele(s)) could then have evolved at a much later stage, seemingly by having advantages of its own, which allowed the *D_i_* allele(s) and various *C_i_* alleles all to be present in the population at the same time, with left-handers in modern proportions. The human tendency to right-handedness and the existence of a smaller proportion of left-handers are therefore entirely separate and logically independent processes, and should not be confused. The present analysis concerns the nature of the *C_i_* alleles, not the origin of the *D_i_* allele(s). It is a plausible alternative evolutionary scenario that the *D_i_* alleles evolved and went to fixation, with all humans having left-hemisphere dominance for language and being right-handed, with a large minority of left-handers never subsequently appearing in human evolution.

## Footnotes

a The assumption of equal frequencies for the *C_i_* alleles is not particularly restricting. We have recalculated the model for 100 loci but with *c_i_* in a triangular distribution from 0.0000508 (1/50 of the 0.00254 for equal *c_i_*) through in equal steps to 0.00508 (twice the equal value *c_i_* of 0.00254). For 1,000,000 replications, the proportions of left-handers in *R* × *R*, *R* × *L*, and *L* × *L* families are 8.47% (8.48%), 16.26% (16.10%), 20.73% (20.28%), the values in parentheses being for equal *ci* from Table 2.
